# How coupled slow oscillations, spindles and ripples coordinate neuronal processing and communication during human sleep

**DOI:** 10.1038/s41593-023-01381-w

**Published:** 2023-07-10

**Authors:** Bernhard P. Staresina, Johannes Niediek, Valeri Borger, Rainer Surges, Florian Mormann

**Affiliations:** 1 Department of Experimental Psychology, University of Oxford, Oxford, UK; 2 Oxford Centre for Human Brain Activity, Wellcome Centre for Integrative Neuroimaging, Department of Psychiatry, University of Oxford, Oxford, UK; 3 Department of Epileptology, University of Bonn Medical Center, Bonn, Germany; 4 Edmond and Lily Safra Center for Brain Sciences, The Hebrew University of Jerusalem, Jerusalem, Israel; 5 Department of Neurosurgery, University of Bonn Medical Center, Bonn, Germany

**Keywords:** Consolidation, Neurophysiology

## Abstract

Learning and plasticity rely on fine-tuned regulation of neuronal circuits during offline periods. An unresolved puzzle is how the sleeping brain, in the absence of external stimulation or conscious effort, coordinates neuronal firing rates (FRs) and communication within and across circuits to support synaptic and systems consolidation. Using intracranial electroencephalography combined with multiunit activity recordings from the human hippocampus and surrounding medial temporal lobe (MTL) areas, we show that, governed by slow oscillation (SO) up-states, sleep spindles set a timeframe for ripples to occur. This sequential coupling leads to a stepwise increase in (1) neuronal FRs, (2) short-latency cross-correlations among local neuronal assemblies and (3) cross-regional MTL interactions. Triggered by SOs and spindles, ripples thus establish optimal conditions for spike-timing-dependent plasticity and systems consolidation. These results unveil how the sequential coupling of specific sleep rhythms orchestrates neuronal processing and communication during human sleep.

## Abstract (web summary)

Using direct recordings from human MTL neurons during sleep, Staresina et al. reveal that neuronal firing and communication—thought to underlie synaptic plasticity and learning—are controlled by coupled slow oscillations, spindles and ripples.

## Main

How are fleeting experiences transformed into durable memories? Sleep constitutes a privileged state for the brain—sheltered from external distractors and tasks—to reorganize and shape neuronal circuits in service of memory formation^1^. Mechanistically, learning and plasticity are governed by two fundamental principles: synaptic consolidation, that is, long-term potentiation of local circuits afforded by short-latency co-firing (spike-timing-dependent plasticity (STDP)), and systems consolidation, that is, the transfer of memory traces across hippocampal–cortical networks via cross-regional communication^2–5^. An unresolved question is how the sleeping brain regulates neuronal (co-)firing rates (FRs) to facilitate these forms of consolidation.

Findings from rodent and human electrophysiological recordings point to a potential role of coupled sleep rhythms, namely slow oscillations (SOs), spindles and ripples, in mediating consolidation processes^6^. SOs reflect fluctuations (<1 Hz) of membrane potentials, toggling between depolarized up-states and hyperpolarized down-states^7,8^. Spindles are waxing-and-waning ~12- to 16-Hz oscillations, generated and sustained through thalamocortical interactions^9^. Ripples are transient high-frequency bursts (~80–120 Hz in humans), best characterized in the hippocampus but more recently also observed in extrahippocampal areas^10,11^. Each of these rhythms has been observed in the human medial temporal lobe (MTL)^12–16^, and their interaction has been linked to behavioral expressions of memory consolidation in rodents^17–19^, with analogous findings in humans being confined to SO–spindle interactions measured with scalp electroencephalography (EEG)^20–23^.

Critically, it remains unclear whether and how the interplay of these three sleep rhythms regulates neuronal activity to support synaptic and/or systems consolidation. What is the division of labor among SOs, spindles and ripples in coordinating local and cross-regional neuronal interactions? In animal models, comprehensive coverage of the MTL and higher-order cortical areas in the same animal and recording session is rare. In humans, data from noninvasive electrophysiological recordings (magnetoencephalography and EEG) are restricted to SOs and spindles, and localization to deeper sources remains challenging. Intracranial EEG (iEEG) in patients with epilepsy allows recording of SOs, spindles and ripples from the MTL and beyond, but is typically confined to field potentials that are only indirectly related to neuronal firing^24^. To overcome these limitations, we recorded from the MTL of human epilepsy patients undergoing presurgical monitoring during natural sleep, using depth electrodes furnished with microwires. These microwires capture neuronal firing (multiunit activity (MUA)), allowing us to assess the role of endogenous sleep rhythms in the regulation of local and cross-regional neuronal activity.

## Results

**Fig. 1:**
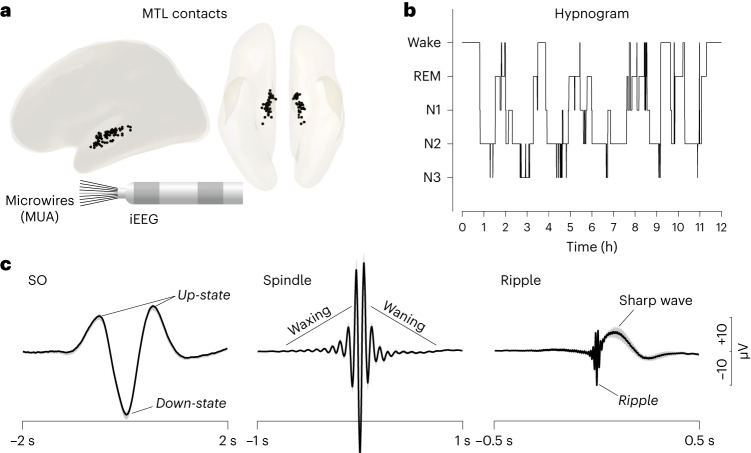
Design. **a**, Locations of iEEG contacts pooled across participants and rendered on a template in MNI space. The inset shows two medial depth electrode contacts (recording the iEEG signal) and the protruding bundle of microwires (recording MUA). **b**, Example hypnogram from a 12-h recording session. Analyses focused on NREM sleep (stages N2 and N3). **c**, Grand average (*n* = 20 sessions recorded from ten individuals, mean ± s.e.m. across sessions) of SOs, spindles and ripples.

We recorded 20 sessions from ten participants (range 1–4 sessions per participant). Depth electrodes were implanted bilaterally, targeting the anterior and posterior hippocampus, amygdala, entorhinal cortex and parahippocampal cortex in all participants (Fig. 1a; for a more detailed visualization of coverage separated by region, see Supplementary Fig. 1). Additional microwires (protruding ~4 mm from the electrode tips) were used to obtain MUA reflecting neuronal firing. Field potentials capturing SOs, spindles and ripples were derived from the most medial macro contacts (after bipolar re-referencing). Unless otherwise stated, data were pooled within each session across these MTL contacts (ten per participant) and corresponding microwires (eight per contact), and statistics were calculated across sessions (*n* = 20). Analyses focused on non-rapid-eye-movement (NREM) sleep (stages N2 and N3; see Fig. 1b for an example hypnogram and Supplementary Table 1 for proportions of sleep stages across sessions). SOs, spindles and ripples were algorithmically detected based on previous methods^25^. Grand averages of the resulting events are shown in Fig. 1c. Note that SO amplitudes are smaller after bipolar re-referencing than after referencing to, for example, linked mastoids; however, morphologies and the number of detected events are comparable across different re-referencing schemes (Supplementary Fig. 2).

### Sequential coupling of SOs, spindles and ripples

**Fig. 2:**
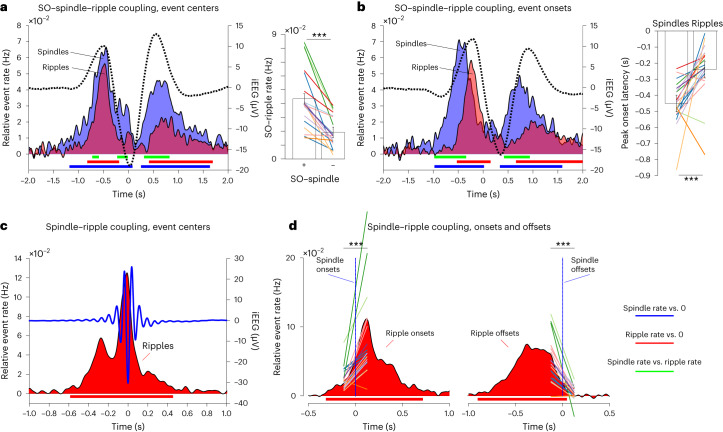
Sequential coupling of SOs, spindles and ripples. **a**, Left, spindle (blue) and ripple (red) rates (center times; left *y* axis) during SOs (dotted black line; right *y* axis), relative to a pre-SO baseline period. Right, SO–ripple rate (ripple centers within ±1 s of SO down-state) as a function of SO–spindle coupling (+, spindle centers within ±1 s of SO down-state; −, no spindles within ±1 s of SO down-state). Bars show means of conditions ± s.e.m. of condition differences. Individual lines represent individual sessions (*n* = 20, recorded from ten individuals), with sessions from the same participants grouped by color. Two-sided paired-samples *t* test: *t*(19) = 6.47, *P* = 3.36 × 10^–6^. **b**, Left, same as **a** but plotting event onsets instead of event centers. Right, latencies (in seconds) of maximal spindle and ripple onset rates from −2 to 0 s relative to SO onset. Bars show means of conditions ± s.e.m. of condition differences. Individual lines represent individual sessions (*n* = 20, recorded from ten individuals), with sessions from the same participants grouped by color. Two-sided paired-samples *t* test: *t*(19) = 4.07, *P* = 6.49 × 10^–4^. **c**, Ripple rates (center times, red; left *y* axis) during spindles (blue line; right *y* axis), relative to a pre-spindle baseline period. **d**, Same as **c** but showing ripple onset rates (red) with respect to spindle onsets (vertical blue line; left) and ripple offset rates (red) with respect to spindle offsets (vertical blue line; right). Individual lines represent individual sessions (*n* = 20, recorded from ten individuals), with sessions from the same participants grouped by color. Ripple rate before versus after spindle onset: *t*(19) = 4.62, *P* = 1.87 × 10^–4^; ripple rate before versus after spindle offset: *t*(19) = 6.57, *P* = 2.75 × 10^–6^ (both two-sided paired-samples *t* tests). Horizontal lines below the event rate plots indicate spindle occurrences versus 0 (blue), ripple occurrences versus 0 (red) and spindle occurrences versus ripple occurrences (green), all *P* < 0.05 (corrected via cluster-based permutation test). ***P < 0.001 (two-sided paired-samples *t* test).

To establish the temporal coupling of SOs, spindles and ripples, we first time-locked spindle and ripple centers to SOs, replicating the finding that both event types are nested in SO up-states (Fig. 2a, left). Importantly, the rate of SO-locked ripples (that is, ripples occurring within ±1 s of an SO down-state) was significantly higher in the presence of a spindle (same time window) than when no spindle was present (*t*(19) = 6.47, *P* < 0.001; Fig. 2a, right). To unravel in more detail the temporal dynamics among SOs, spindles and ripples, we repeated the analysis using event onsets instead of event centers. As shown in Fig. 2b (left), spindle onsets increased in earlier phases of the SO up-state than did ripple onsets. Indeed, the maximum event rate (within −2 to 0 s relative to the SO down-state onset) occurred, on average, at −451 ms for spindles and at −241 ms for ripples (*t*(19) = 4.07, *P* < 0.001).

To directly test whether spindles might increase the likelihood for ripples to occur, we extracted the occurrence of ripple centers, onsets (start times) and offsets (end times) with respect to spindle centers, onsets and offsets. Figure 2c confirms the significant increase in ripple rates around spindle centers, revealing a tendency for ripples to occur before the spindle center (that is, during the ‘waxing’ spindle phase; *t*(19) = 5.04, *P* < 0.001, −1 to 0 s versus 0 to 1 s). The onset- and offset-locked analysis shown in Fig. 2d then corroborated that most ripples coupled to spindles begin after spindle onset and end before spindle offset. This observation was further confirmed by directly comparing ripple rates in a 250-ms window before spindle onset versus 250 ms after spindle onset (*t*(19) = 4.62, *P* < 0.001) and ripple rates in a 250-ms window before spindle offset versus 250 ms after spindle offset (*t*(19) = 6.57, *P* < 0.001). For ripple-locked rates of SOs and spindles, see Supplementary Fig. 3. Together, these results show that spindles and ripples cluster in the SO up-state, with spindles increasing the probability for ripples to occur between their start and end times.

### Neuronal FRs increase across SOs, spindles and ripples

**Fig. 3:**
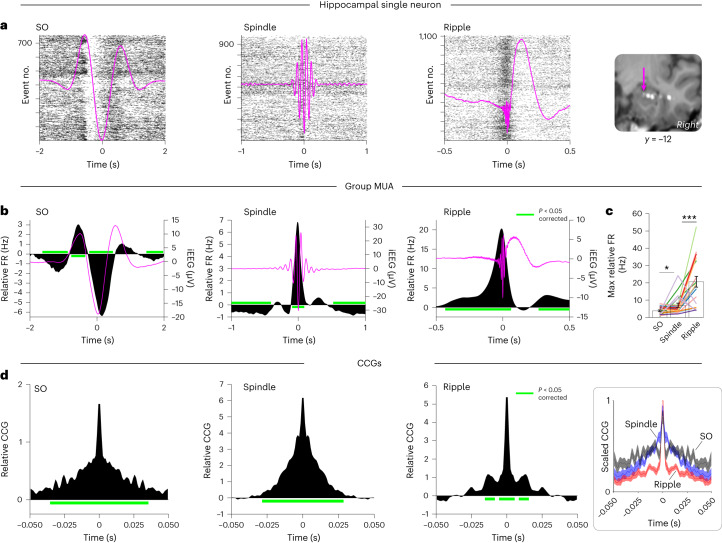
Modulation of neuronal (co-)FRs by SOs, spindles and ripples. **a**, Single-participant, single-neuron example. Raster plots show action potentials across time (*x* axis) for individual events (*y* axis), with the mean SO, spindle and ripple event-related potentials (ERPs) of the session superimposed (magenta). ERPs were band-pass filtered in the SO and spindle detection range, and from 0.1 to 120 Hz for ripples, to preserve the sharp-wave component for visualization. Right, magnetic resonance imaging–computed tomography scan (in MNI space) depicting the macro contact and the microwire bundle (arrow) from which this single unit was isolated. **b**, MUA FRs relative to pre-event baselines (black; left *y* axis), averaged across sessions for SOs (left), spindles (middle) and ripples (right) (*n* = 20, recorded from ten individuals; see Supplementary Fig. 5 for mean ± s.e.m.). Grand-average ERPs across sessions are superimposed (magenta; right *y* axis). **c**, Maximal FRs per session (*n* = 20, recorded from ten individuals), illustrating the stepwise increase across SOs, spindles and ripples. Bars show means ± s.e.m. of conditions. Individual lines represent individual sessions, with sessions from the same participants grouped by color. SOs versus spindles: *t*(19) = 2.21, *P* = 0.040; spindles versus ripples: *t*(19) = 3.96, *P* = 8.45 × 10^–4^ (both two-sided paired-samples *t* tests). **d**, Event-locked cross-correlograms (CCGs) of neuronal firing among pairs of microwires, relative to shift predictors and non-event surrogates. Right, CCGs for SOs (black), spindles (blue) and ripples (red) scaled between 0 and 1 (mean ± s.e.m. across sessions; *n* = 20, recorded from ten individuals), illustrating the stepwise narrowing of co-firing windows. Horizontal green lines indicate FRs versus 0, *P* < 0.05 (corrected via cluster-based permutation test). *P < 0.05, ***P < 0.001 (two-sided paired-samples *t* test).

We next turned to the question of whether and how neuronal FRs are modulated by SOs, spindles and ripples. Note that previous studies in humans have reported modulation of FRs by SOs^26^, spindles^27,28^ and (wake) ripples^29^, but those FRs have not previously been examined side by side in the same participants, brain regions and behavioral states (for example, sleep). As shown in Fig. 3a (single-neuron example from the hippocampus) and 3b (MUA pooled across contacts and averaged across sessions), all three event types modulated FRs relative to pre-event baseline intervals, but in different manners. During SOs, FRs showed an increase during the up-state and a marked decrease during the down-state, pointing to an active silencing function of SO down-states (FRs below baseline levels). During spindles, FRs increased around the spindle centers and decreased 500 ms before and after the centers, likely reflecting the effect of coupled SO down-states (Fig. 2a and Supplementary Fig. 5). Finally, FRs showed a pronounced increase during ripples, exhibiting a ~500-ms ramp-up period before the ripple start. To quantify the stepwise increase in FRs across SOs, spindles and ripples, we derived, for each session, the maximum FRs within ±2 s of the three event centers. As shown in Fig. 3c, there was a significant increase in maximum FRs from SOs to spindles (*t*(19) = 2.21, *P* = 0.040) and from spindles to ripples (*t*(19) = 3.96, *P* < 0.001). Note that we chose our pre-event baseline intervals based on previous work^15^, but results remained unchanged when using different intervals or matched non-event surrogates (Supplementary Fig. 4).

### Neuronal co-firing during SOs, spindles and ripples

As mentioned above, one central mechanism driving learning-related changes in cell assemblies is short-latency co-firing, capable of inducing long-term potentiation via STDP^30^. A recent report showed that spindles in the lateral temporal cortex group co-firing between neurons within 25 ms^28^, but how this effect relates to potential co-firing during SOs and ripples is unclear. To examine co-firing patterns during SOs, spindles and ripples, we derived cross-correlograms (CCGs) in ±50-ms windows centered on event maxima. CCGs were calculated for all pairwise combinations of microwires in a given bundle (resulting in symmetrical CCGs), including only wires that showed a minimum FR of 1 Hz across all NREM sleep. Resulting CCGs were corrected in two steps. First, we subtracted ‘shift predictor’ CCGs, reflecting the cross-correlation of wire 1 FRs during event *n* with wire 2 FRs during event *n* + 1, thereby accounting for the overall FRs during a particular event type^31^. Second, we subtracted CCGs derived from matched non-event surrogates (also shift-predictor-corrected). In the resulting CCGs, values greater than zero thus signify highly event-specific co-firing in local assemblies. As shown in Fig. 3d, all three event types elicited significant neuronal co-firing. Importantly, however, the temporal windows of co-firing showed a stepwise decrease across SOs, spindles and ripples. Significant co-firing spanned a range of 35 ms for SOs, 28 ms for spindles and 5 ms for ripples, with a second peak between ~9 and 15 ms (reflecting an oscillatory cycle at ~70–110 Hz). The stepwise narrowing of CCGs is further highlighted in Fig. 3d (right), where all three CCGs were scaled between 0 and 1 within each session. Together, these findings suggest that ripples are most apt in creating conditions conducive to STDP.

### Relationship between FRs and event occurrences

**Fig. 4:**
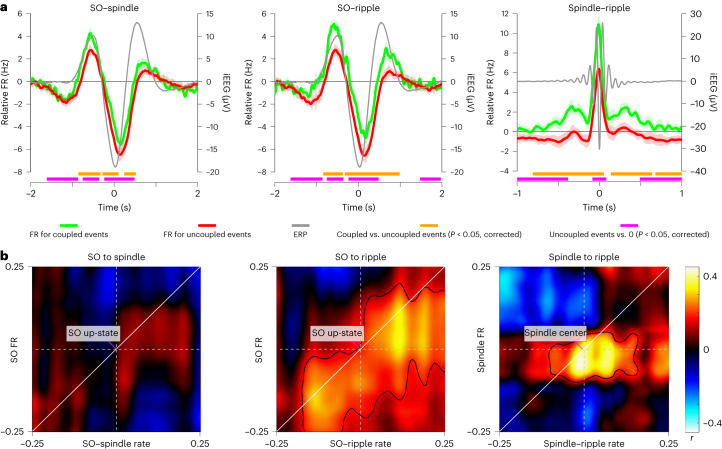
Relationship between neuronal FRs and event occurrences. **a**, FRs as a function of event contingencies (target event center within ±1 s of seed event center). Green and red lines represent mean baseline-corrected FRs across sessions for coupled versus uncoupled events ± s.e.m. of condition differences (*n* = 20 sessions, recorded from ten individuals; left *y* axis). Gray lines represent grand-average ERPs of seed events (irrespective of target presence; right *y* axis). Note that FRs are elevated for all coupled events, but SOs and spindles without ripples also elicit significant above-baseline FRs. Horizontal orange and magenta lines indicate *P* < 0.05 (corrected via cluster-based permutation test). **b**, Time-by-time correlations between seed event FRs and target event occurrences. Correlations (Pearson’s *r*) are shown for 500-ms intervals between SO FRs and SO-locked spindle rates (left), SO FRs and SO-locked ripple rates (middle) and spindle FRs and spindle-locked ripple rates (right). Below diagonal: FRs precede event occurrences; above diagonal: FRs follow event occurrences. Contour, *P* < 0.05 (corrected via cluster-based permutation test).

The strong increase in FRs during ripples (Fig. 3b) raises the question of whether SO- and spindle-related FRs might merely reflect ripple-related FRs, given that ripples are coupled to SOs and spindles (Fig. 2a). Conversely, genuine FR increases during SOs and spindles might trigger ripple occurrences by mediating the observed ramp-up preceding ripples (starting ~500 ms before ripple centers; Fig. 3b). To adjudicate between these two scenarios, we first conducted the event-locked FR analysis again, but separated event types of interest (for example, spindles, ‘seed’) based on the presence or absence of another event type (for example, ripples, ‘target’). Presence or absence was coarsely defined based on the algorithmic detection of target event centers occurring within ±1 s of the seed event center. As shown in Fig. 4a, both SOs and spindles had enhanced FRs when ripples were present (and vice versa; Supplementary Fig. 5). Importantly, however, FRs in the SO up-states and around spindle centers also exhibited significant increases when no ripples were present (for all pairwise seed–target combinations, see Supplementary Fig. 5). This result indicates that ripples are not the (sole) driver of FR increases during SOs and spindles.

To address the second question, that is, whether SO- and spindle-related FRs might predict ripple occurrences, we computed, separately for each session, a time-by-time correlation between seed event FRs and target event occurrences across the ten MTL contacts of each participant. In other words, does a contact that shows greater FRs during spindles also show greater spindle-locked ripple rates? The resulting correlation maps (*n* = 20) were then tested against zero via nonparametric cluster permutation tests. As shown in Fig. 4b, we observed no association between FRs during SO up-states and SO-locked spindle rates. However, FRs during SO up-states were positively correlated with SO-locked ripple rates. This relationship was even more pronounced for FRs around spindle centers and spindle-locked ripple rates. The narrower window of modulation during spindles likely reflects the more transient increase in FRs (~100 ms) compared to SO up-states (~500 ms; Fig. 3b). Critically, significant correlations were seen below the diagonal, that is, earlier SO and spindle FRs predicted later ripple rates. Together, these results suggest that the emergence of ripples is, at least in part, facilitated by gradual increases in FRs afforded by SOs and spindles. For example raw traces showing coupled and uncoupled ripples, spindles and SOs alongside FRs, see Supplementary Fig. 6a,b.

### MTL network dynamics linked to SOs, spindles and ripples

**Fig. 5:**
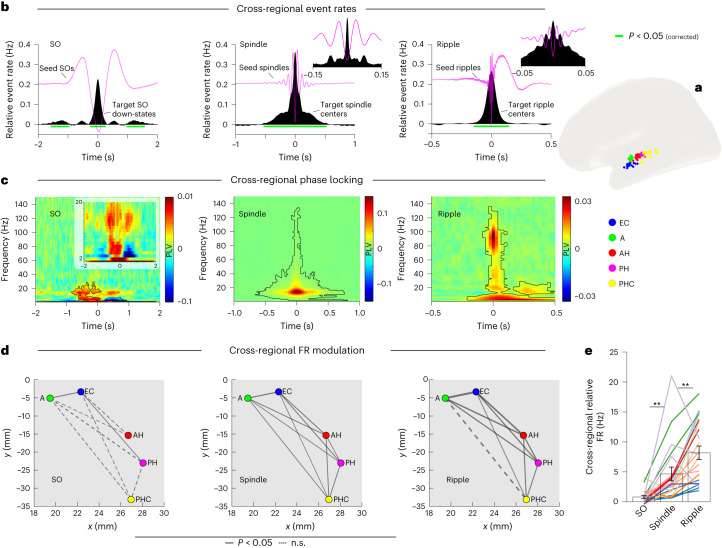
Network dynamics. **a**, Locations of the anterior hippocampus (AH, red), posterior hippocampus (PH, magenta), amygdala (A, green), entorhinal cortex (EC, blue) and parahippocampal cortex (PHC, yellow). MNI coordinates were averaged across participants and projected onto the left hemisphere of a standard template. **b**, Cross-regional event rates relative to a pre-event baseline window, showing significant co-occurrences of SOs, spindles and ripples. Insets show data after applying 10-ms instead of 100-ms smoothing, highlighting the temporal precision of co-occurrences during spindles and ripples. Horizontal green lines indicate *P* < 0.05 (corrected via cluster-based permutation test). **c**, Time- and frequency-resolved phase locking values (PLVs) between a seed region and four same-hemisphere target regions, relative to a pre-event baseline and averaged across sessions (*n* = 20, recorded from ten individuals). Contour, *P* < 0.05 (corrected via cluster-based permutation test). **d**, Cross-regional event-locked FRs for SOs, spindles and ripples (averaged across a 100-ms window centered on the event maximum). Nodes reflect projections of MTL coordinates (MNI space) in the *xy* plane. Edge widths reflect effect sizes (*t* values of paired-samples *t* test) of bidirectional increases in FRs relative to a pre-event baseline. Solid lines indicate significant bidirectional upregulation of FRs (*P* < 0.05, two-tailed, uncorrected). Dashed lines indicate an absence of significance. **e**, All edges collapsed. Bars show mean ± s.e.m. of conditions. Individual lines represent individual sessions (*n* = 20, recorded from ten individuals), with sessions from the same participants grouped by color. SOs versus spindles: *t*(19) = 3.35, *P* = 0.003; spindles versus ripples: *t*(19) = 2.85, *P* = 0.01 (both two-sided paired-samples *t* tests). **P ≤ 0.01 (two-sided paired-samples *t* test).

Lastly, systems consolidation relies on interregional information transfer beyond local cell assemblies^32,33^. We thus set out to examine the role of SOs, spindles and ripples in cross-regional interactions among the separate MTL regions targeted in our recordings (anterior hippocampus, posterior hippocampus, amygdala, entorhinal cortex and parahippocampal cortex; Fig. 5a). Note that bipolar referencing was used for this dataset, thereby mitigating the risk of spurious effects reflecting volume conduction.

In a first step, we assessed the extent of cross-regional event occurrences, that is, the likelihood of, for example, a ripple also occurring in region B (target region) when a ripple is detected in region A (seed region). All combinations of same-hemisphere contact pairs were collapsed (resulting in symmetrical histograms), and target event rates were again compared to a pre-event baseline interval. As shown in Fig. 5b, all three event types were coupled across MTL regions. It is worth highlighting the precise cross-regional locking to the seed events for spindles and ripples (insets), indicating that the majority of other spindle and ripple centers occurred either in the same or the next cycle, thereby optimizing conditions for mutual communication^34^.

To corroborate and expand on the finding of cross-regional communication, we calculated pairwise phase-locking values (PLVs), reflecting consistent phase differences of two regions across events^35^. This was done in a time- and frequency-resolved manner, centered around SOs, spindles and ripples, and statistically compared to pre-event baselines (again collapsing across same-hemisphere contact pairs). As shown in Fig. 5c, this analysis confirmed that all three event types elicited significant PLVs in the seed event’s frequency range (see previous analysis). Interestingly, regions also coupled in the spindle band during SOs and following ripples, extending a recent finding that spindles mediate hippocampal–cortical communication during ripples^25^. For all pairwise co-occurrences and PLVs across regions, see Supplementary Fig. 7.

As a last step, we assessed the extent to which the observed functional connectivity translates to cross-regional modulation of neuronal FRs, for example, a ripple in region A leading to an increase in FR in region B. Specifically, we derived, for each pair of same-hemisphere MTL contacts, the baseline-corrected FRs in the target region (averaged across a 100-ms window centered on the seed region’s event maximum). Resulting connection strengths across the MTL (*t* values combined across both seed and target directions for a given pair and across both hemispheres) are shown in Fig. 5d. Collapsing all connection edges illustrates the stepwise increase in cross-regional FR modulation across SOs, spindles and ripples (Fig. 5e). All pairwise combinations of seed event–target FRs separated by region and hemisphere are shown in Supplementary Fig. 8.

Taken together, these results reveal that SOs, spindles and ripples are functionally coupled across MTL regions. This coupling leads to fine-tuned network modulation of neuronal FRs (most strongly during ripples), putatively well suited to support systems-level consolidation.

## Discussion

Our findings elucidate the mechanisms through which SOs, spindles and ripples coordinate neuronal FRs and communication during sleep, establishing conditions conducive to synaptic and systems consolidation. Although recent work has converged on the importance of these sleep rhythms’ (co-)occurrence for memory consolidation^6,36^, their division of labor in the process has remained elusive. Our data suggest that SO up-states first establish a coarse time window for spindles and ripples to coincide, consistent with previous reports of triple nesting of these events^12–15,17–19^. Importantly, spindles enhance the likelihood (Fig. 2a) and set a more fine-grained temporal frame for ripples to occur (Fig. 2c), akin to a relay function of spindles between SOs and ripples. Mechanistically, our data suggest that SO up-states and, to a greater extent, the waxing phase of spindles elevate FRs to a threshold at which ripples are triggered (Fig. 4b and Supplementary Fig. 6). This results in an exponential increase in FRs (Fig. 3b,c) and concomitant synchronization in local cell assemblies (Fig. 3d) and across the MTL (Fig. 5).

The enhancement of neuronal FRs and ripple rates when SOs and spindles are coupled (Figs. 2a and 4a and Supplementary Figs. 5 and 6) dovetails with a series of recent findings linking SO–spindle coupling to physiological and behavioral manifestations of memory consolidation^20–22,37,38^. Moreover, optogenetic enhancement of spindles in mice was found to elicit hippocampal ripples and memory improvements, particularly when stimulation occurred during SO up-states^17^. That said, the facilitation of ripples is unlikely to be the sole function of SOs and spindles. Likewise, SOs and spindles are clearly not the only means through which ripples can be triggered; for one, ripples are readily observed during waking states^11,39,40^. However, in the absence of external input and conscious control, coupled SO–spindle events constitute a controlled yet effective mechanism of gradually elevating neuronal FRs and thereby triggering ripples^41,42^.

Apart from grouping spindles and ripples in their up-states, one striking feature of the SO-locked analysis was the active inhibition of FRs (below baseline levels) during down-states (Fig. 3b). This effect (also referred to as OFF periods) is well documented across species^8,26,43,44^ and points to a dynamic alternation between active consolidation processes during up-states and homeostatic recalibration and/or pruning of irrelevant circuits during down-states^45,46^.

Spindle–ripple coupling has been established in animal and human intracranial recordings^12,14,15,25,41,47,48^. A consistent finding in these (and our current) data is that spindles nest ripples after their onset and just before their maximum^15,19,49^. In light of the ability of spindles to synchronize wide-ranging neuronal networks^9,50,51^, an intriguing possibility is that spindles not only drive ripple emergence during their waxing phase but also support the interregional transfer of information reactivated during ripples via the ongoing synchronization during their waning phase^36^. Indeed, we observed increased spindle-band phase locking after ripples across the MTL (Fig. 5c), extending our recent finding of post-ripple coupling between hippocampal and scalp recordings^25^. Although evidence for ripple-mediated memory reactivation during sleep is still lacking in humans, it has been firmly established in rodents^10,52–55^.

Memory consolidation ultimately reflects adaptive changes in brain structure and function^2,4^. On a synaptic level, such changes can be afforded by long-term potentiation and STDP, elicited by short-latency co-firing of participating neurons^56–58^. Ripples reflect a surge in local circuit synchronization, ideally poised to induce such synaptic changes^10,59^. Our results reveal that, relative to SOs and spindles, ripples indeed create the narrowest (<10 ms) time windows of neuronal co-firing (Fig. 3d), thus supporting the notion that ripples are a viable mechanism to induce synaptic consolidation in humans.

Finally, the persistence of memories is thought to rely on their distribution across hippocampal–cortical networks (‘systems consolidation’)^2,32,33,36,60,61^. In rodents and nonhuman primates, ripples have been shown to influence the activation in and connectivity among long-range cortical and subcortical brain networks^62,63^. Although we observed that SOs, spindles and ripples are all synchronized across the MTL (Fig. 5a,b), cross-regional modulation of FRs was again strongest during ripples (Fig. 5c). Together, these results suggest that, whereas SOs and particularly spindles open and maintain channels for cross-regional communication, ripples provide further means to effectively forge local and brain-wide functional networks.

Although our results are consistent with a role of SO–spindle–ripple coupling in promoting synaptic and systems consolidation, a clear shortcoming of the current study is the lack of a proper assay to capture behavioral expressions of memory consolidation. Devising such an assay is a daunting challenge in patient work, as robust conclusions ideally require not only large sample sizes to allow cross-participant correlations but also multiple sessions within a participant to compare nights of ‘better’ consolidation to nights of ‘worse’ consolidation. That said, a recent iEEG study showed that stimulating the prefrontal cortex during SO up-states in the MTL augmented cross-regional SO–spindle–ripple coupling and improved recognition memory in human participants^64^. Another caveat is that the raw numbers of event (co-)occurrences as derived here are somewhat arbitrary, as they heavily rely on the parameters set in the detection algorithms. For instance, we have shown previously^15^ that, for example, the proportion of SO-triggered spindles that also contain ripples increases from 6% to 10% when minimally relaxing the detection thresholds (setting the ripple detection threshold from the top 1% to the top 2% amplitude). However, examination of relative event rates after normalization to a pre-event baseline or to matched surrogate events is still highly informative, as the detection parameters (and ensuing miss and/or false alarm rates) are held constant across target and baseline (or control) epochs. By the same token, the ‘absence’ of a co-occurring event (Fig. 4a and Supplementary Fig. 5) warrants interpretive caution, as subthreshold events might have been missed by the detection procedure.

To conclude, we show that SOs, spindles and ripples systematically interact to coordinate neuronal FRs and communication during NREM sleep. Ignited by SO up-states, spindles increase the likelihood for ripples to occur. In turn, ripples lead to a surge in neuronal firing and drive short-latency coactivation in local assemblies, fostering conditions permissive of STDP and long-term potentiation. Finally, sleep rhythms are synchronized across the MTL, facilitating cross-regional neuronal communication thought to underlie systems consolidation.

## Methods

### Participants and recordings

Macro and simultaneous microwire recordings were performed over 20 sessions in ten participants (range 1–4 sessions per participant) undergoing invasive presurgical seizure monitoring for the treatment of medically refractory epilepsy (five men, five women, all right-handed, mean age 39.9 years (range 20–62 years)). No statistical methods were used to predetermine sample sizes, but our sample size is similar to those reported in previous publications^12,15,16,28^. The study was approved by the Medical Institutional Review Board at the University of Bonn, and participants provided written informed consent. No financial compensation was provided for participation. Depth electrodes were implanted bilaterally, targeting the anterior and posterior hippocampus (hippocampal head and body, respectively), amygdala, entorhinal cortex and parahippocampal cortex in all participants (Fig. 1a and Supplementary Fig. 1). Depth electrodes were furnished with bundles of nine microwires each (eight high-impedance recording electrodes and one low-impedance reference, AdTech) protruding ~4 mm from the electrode tips. The differential signal from the microwires was amplified using an ATLAS system (Neuralynx), filtered between 0.1 and 9,000 Hz and sampled at 32 kHz.

MUA reflecting neuronal firing was obtained from these microwires by using the Combinato package^65^. In brief, spikes were independently identified from each wire via a thresholding procedure and extracted after band-pass filtering (300–3,000 Hz). Combinato’s default procedure for artifact removal was applied: 500-ms time bins containing >100 events were excluded; events exceeding an amplitude of 1 mV were removed; 3-ms time bins with coinciding events in >50% of all channels were excluded. Remaining spikes were spike sorted, and artifact clusters were identified. Combinato’s default parameters were used in each step. In previous work, we showed that simultaneous spikes on multiple wires tended to occur in clusters classified as artifacts, whereas, in events classified as MUA, only 3.4% stemmed from duplicate spikes^66^. All nonartifact clusters were then separately merged in each channel. All FR analyses reported here were derived from these MUA signals. Single-unit activity is shown in Fig. 3a for illustrative purposes only.

Field potentials capturing SOs, spindles and ripples were derived from the proximal (most medial) macro contacts, thus mitigating contamination of the iEEG signal by high-frequency action potentials^67^. Each macro contact was re-referenced in a bipolar fashion, subtracting the signal from the neighboring contact on the same electrode. This procedure was chosen to safeguard against signal spread from adjacent MTL areas, which would be a particular concern for connectivity analyses. The effect of different referencing schemes (bipolar, white matter contact, linked mastoids) on the resulting event numbers and morphologies is shown in Supplementary Fig. 2. Event densities separated by MTL region are shown in Supplementary Fig. 9.

For polysomnography, additional surface electrodes were applied according to the 10–20 system alongside electrooculography and electromyography electrodes. Sleep staging was performed according to American Academy of Sleep Medicine guidelines^68^, and all analyses were confined to NREM sleep (stages N2 and N3). Figure 1b shows an example hypnogram, and proportions of sleep stages across sessions are listed in Supplementary Table 1. The number of minutes spent in NREM sleep and the number of detected events are shown separately for each session in Supplementary Table 2. For coupling and FR analyses separated by NREM sleep stage (N2 and N3), see Supplementary Fig. 10.

### Artifact rejection and event detection

Data were analyzed in MATLAB (version R2022a) using FieldTrip (version 20201229) functions^69^. Before sleep event detection, each contact was subjected to preprocessing and artifact detection. Preprocessing included downsampling to 1 kHz, removing 50 Hz of line noise and harmonics up to 200 Hz via ±1-Hz band-stop filters and 0.1-Hz high-pass filtering to remove slow signal drifts. The iEEG signal was inverted so that positive peaks reflect up-states. For artifact detection, two copies of the raw signal were created: one after applying a 250-Hz high-pass filter and one after taking the first derivative of the data (reflecting signal gradients). The three signals (raw, high-pass filtered, gradient) were *z* scored within each sleep stage, and a data point was classified as artifactual if it exceeded a *z* score of 6 in any one of the three signals or a *z* score of 4 in the raw signal as well as in any of the two other signals (high-pass filtered, gradient). Artifacts <3 s apart were merged, and artifactual samples were additionally padded by 1 s on each side (Supplementary Fig. 11).

SOs, spindles and ripples were algorithmically detected based on previous methods^15,25^. In brief, for SO detection, the signal was first band-pass filtered between 0.3 and 1.25 Hz. Second, all zero crossings were determined in the filtered signal, and event duration was determined for SO candidates as the time between two successive positive-to-negative zero crossings (that is, a down-state followed by an up-state). Events that lasted between 0.8 and 2 s entered the next step. Third, event amplitudes were determined for the remaining SO candidates (trough and trough-to-peak amplitude). Events in which both amplitudes exceeded the mean plus 1 s.d. of all candidate events were considered SOs. SO-locked analyses were based on either the event onset (position of positive-to-negative zero crossing or up-state to down-state transition), the event center (maximal trough after the onset, that is, down-state) or the event maximum (maximal peak after the onset, that is, up-state).

For spindle detection, the signal was band-pass filtered at 12–16 Hz, and the root mean square (RMS) signal was calculated based on a 200-ms window followed by an additional smoothing with the same window length. A spindle event was identified whenever the smoothed RMS signal exceeded a threshold, defined by the mean plus 1 s.d. of the RMS signal across all NREM data points, for at least 0.4 s but not longer than 3 s. Time points exceeding an upper threshold determined by the mean RMS signal plus nine times its s.d. were excluded. Lastly, spindles were required to exhibit a minimum of six cycles in the raw iEEG signal. The onset (start) and offset (end) of spindles were defined as the upward and downward threshold crossings, respectively, of the smoothed RMS signal. Unless otherwise noted, spindle centers were defined as the maximal trough.

Detection of ripples followed the same procedure, except that the iEEG signal was band-pass filtered from 80 to 120 Hz and both RMS calculation and smoothing were based on 20-ms windows. Detection and upper cutoff thresholds were defined by the mean of the RMS signal plus three and nine times the s.d., respectively. Potential ripple events with a duration of <38 ms (corresponding to three cycles at 80 Hz) or >200 ms were rejected. Additionally, all ripple events were required to exhibit a minimum of three cycles in the raw EEG signal. Unless otherwise noted, ripple centers were defined as the maximal trough. For example traces of interictal epileptiform discharges versus ripples detected by our algorithms, see Supplementary Fig. 11.

### Anatomy

Postimplantation magnetic resonance imaging–computed tomography scans were available for nine of the ten participants. Scans were normalized to the Montreal Neurological Institute (MNI) space using SPM12. Contact locations were then manually identified to create group-level representations of target locations (Fig. 1a). To visualize the MTL network (Fig. 5), *xyz* coordinates were averaged across participants. For a more detailed visualization of macro contacts shown in Supplementary Fig. 1, a 3-mm-radius sphere was placed on contact coordinates. For sagittal views, all *y* and *z* coordinates were projected on the mean *x* coordinate across participants. For coronal views, all *x* and *z* coordinates were projected on the mean *y* coordinate across participants. Percentage coverage refers to the number of participants with target spheres at a given voxel.

### Analyses and statistics

Analyses of event occurrences and FRs were based on peri-event time histograms, with 1-ms bin sizes. Event rates and FRs were converted to hertz and temporally smoothed with a 100-ms Gaussian kernel unless otherwise noted. Resulting histograms were then corrected to pre-event baseline intervals (−2.5 to −2 s for SOs and spindles, −1.5 to −1 s for ripples^15^). For the CCG analysis, additional non-event surrogates were derived. For instance, for each participant’s *n* observed ripple events, we derived *n* nonripple events, that is, artifact-free NREM epochs matching the duration of each individual event including an additional padding of 1.5 s before and after which our ripple detection algorithm did not indicate the presence of a ripple (irrespective of the presence or absence of spindles or SOs). Furthermore, to ensure that signal properties were maximally matched between target events and surrogates, surrogates were drawn only from a 10-min time window before and after the corresponding ripple event. The probability underlying the randomized selection of surrogate events within such a 10-min interval was modulated according to a normal distribution. Epochs once assigned to surrogate events were discarded from subsequent iterations to exclude overlapping surrogates.

For each session, analyses were performed separately for each MTL contact, pooling FRs across the eight microwires in case of MUA analyses (except for the CCG analysis shown in Fig. 3d in which pairwise cross-correlations of FRs were examined across microwires). Session-specific results were obtained by averaging results across contacts (except for cross-contact correlations shown in Fig. 4c and connectivity analyses shown in Fig. 5), weighing each contact by the number of contributing events of that contact. Final statistical analyses were performed across sessions (*n* = 20). We chose not to average multiple sessions obtained from the same participant, as the average temporal gap between successive sessions was 4 days (range 1–8 days). Importantly, even small shifts of microwires (‘micromovements’) would lead to a different composition of MUA across sessions. That said, our results also hold when collapsing multiple sessions per participant (with a new *n* of 10). For SO-locked coupling, there was a significant increase in spindles and ripples in the up-state (−750 to −250 ms, both *t*(9) > 6.54, *P* < 0.001). For spindle-locked coupling, there was a significant increase in ripples in a 100-ms window centered on the spindle (*t*(9) = 4.15, *P* = 0.003). Likewise, SO-locked FRs were significantly reduced in the SO down-state (−250 to 250 ms, *t*(9) = 5.41, *P* < 0.001) and increased in a 100-ms window centered on spindles (*t*(9) = 4.34, *P* = 0.002) and ripples (*t*(9) = 4.50, *P* = 0.001).

For the CCG analysis shown in Fig. 3d, we used FieldTrip’s ft_spike_xcorr function (obtaining both the CCG and the shift predictor as a control condition). Time windows to derive CCGs were equated across events and set to 150 ms centered on event maxima (to capture the SO up-state), including positive and negative lags up to 50 ms. Bin size was 1 ms, and resulting CCGs were smoothed with a 5-ms Gaussian kernel.

For the cross-regional PLV analysis shown in Fig. 5, time–frequency representations were extracted centered on target event maxima (using FieldTrip’s mtmconvol function) for frequencies from 1 to 150 Hz in steps of 1 Hz, using a sliding Hanning-tapered window advancing in 25-ms steps. The window length was frequency dependent, such that it always comprised a full number of cycles, but at least five cycles and at least 100 ms, ensuring reliable phase estimates for higher frequencies for which five cycles would result in windows that were too short.

To correct statistical analyses for multiple comparisons, a cluster-based permutation procedure was applied as implemented in FieldTrip, using 1,000 permutations, a cluster threshold of *P* < 0.05 and a final threshold for significance of *P* < 0.05 (all two-tailed). Data distribution was assumed to be normal, but this was not formally tested.

### Reporting summary

Further information on research design is available in the Nature Portfolio Reporting Summary linked to this article.

## Online content

Any methods, additional references, Nature Portfolio reporting summaries, source data, extended data, supplementary information, acknowledgements, peer review information; details of author contributions and competing interests; and statements of data and code availability are available at 10.1038/s41593-023-01381-w.

## Supplementary information

Supplementary Information Supplementary Figs. 1–11 and Tables 1 and 2.

Reporting Summary

## Data Availability

Data and analysis scripts to reproduce the main results are shared on the Open Science Framework (https://osf.io/8kevm/).
