# Supplementary material for: How coupled slow oscillations, spindles and ripples coordinate neuronal processing and communication during human sleep
Source: Nat Neurosci. 2023 Jul 10;26(8):1429–37. doi: 10.1038/s41593-023-01381-w (PMC10400429; doi:10.1038/s41593-023-01381-w)
Supplement: Supplementary file 1 — Supplementary Figs. 1–11 and Tables 1 and 2. [file 41593_2023_1381_MOESM1_ESM.pdf]

nature neuroscience

Article https://doi.org/10.1038/s41593-023-01381-w

How coupled slow oscillations, spindles and ripples coordinate neuronal processing and communication during human sleep

In the format provided by the authors and unedited

Supplemental results

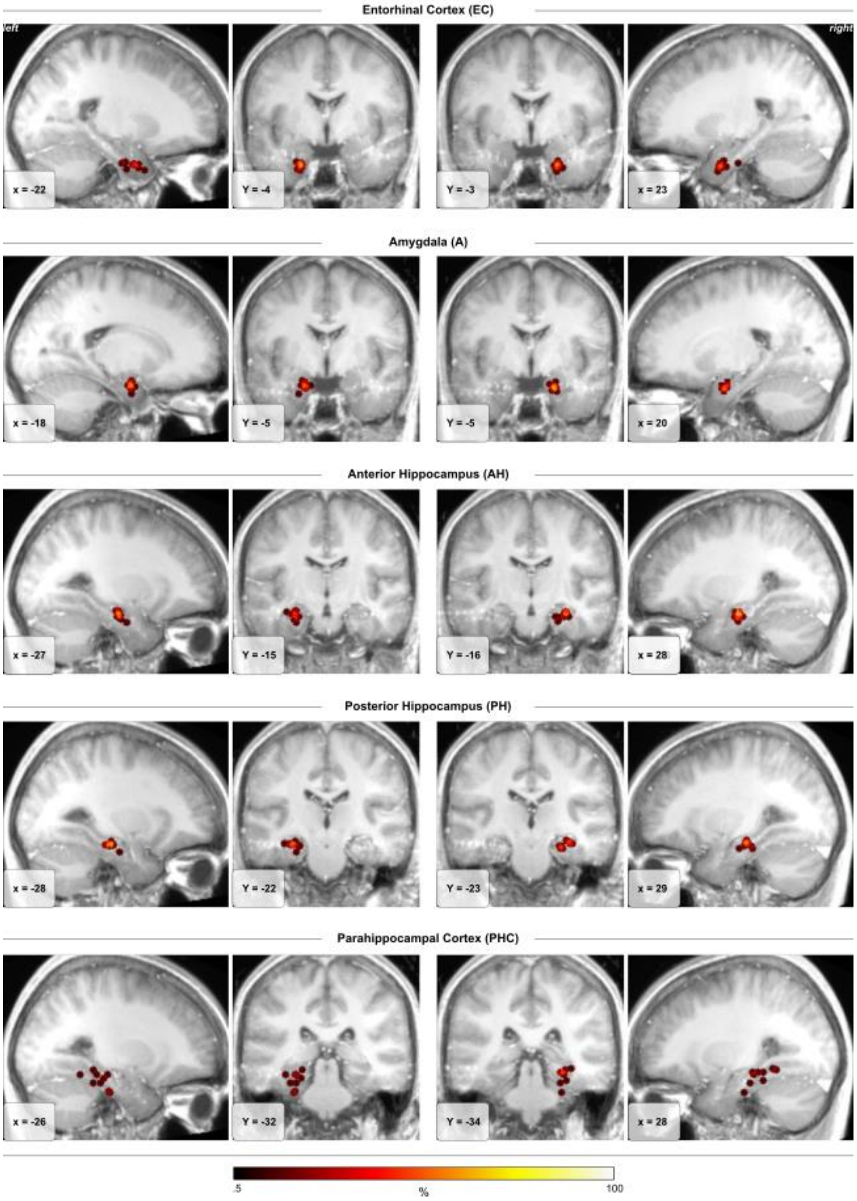

Figure S1. Locations of macro MTL contacts, projected on the mean T1 scan across participants. Heatmaps show coverage across participants after placing a 3 mm-radius sphere on each electrode centre. For sagittal views, all participants' coordinates are projected onto the average x coordinate per region. For coronal views, coordinates are projected onto the average y coordinate per region. Coordinates refer to MNI space.

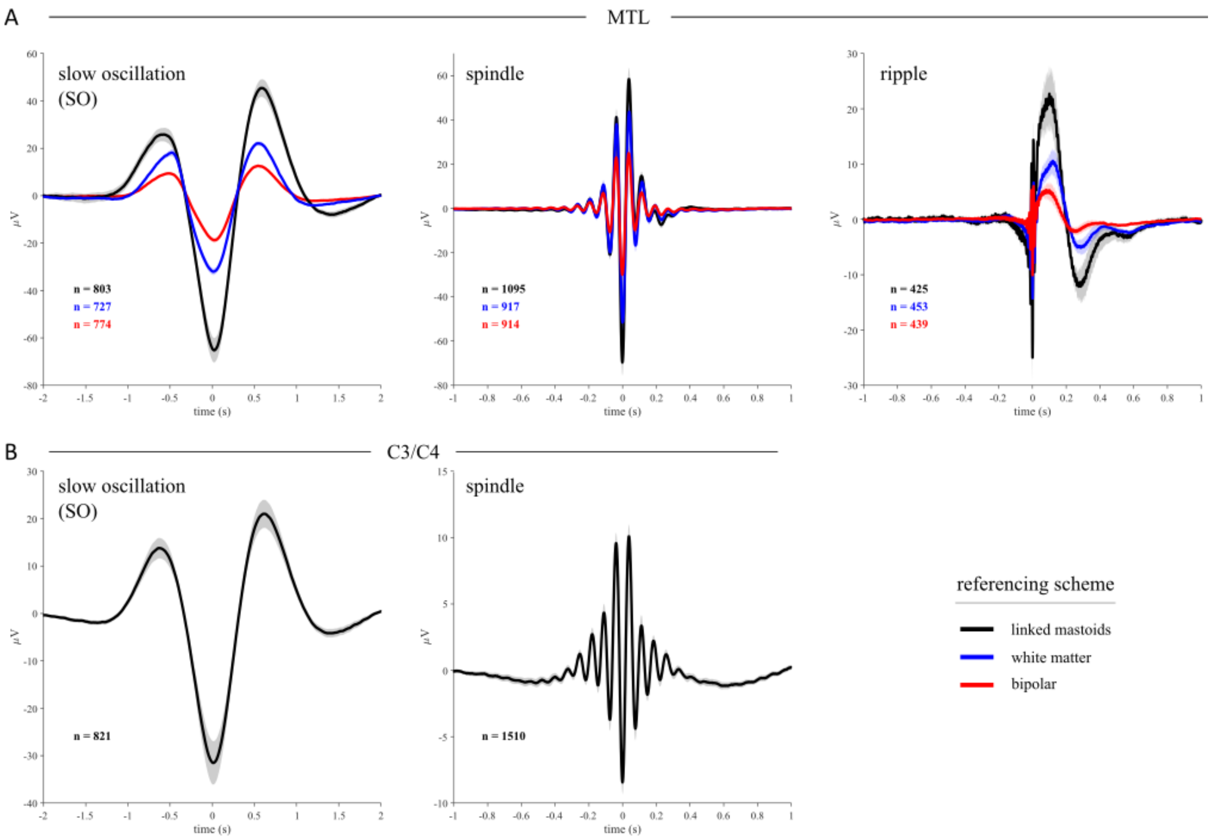

Figure S2. Effect of different referencing schemes on ERP amplitudes. Grand averages (N=20 sessions recorded from 10 individuals) ± SEM are shown, including the average numbers of detected events. **A**. Comparison of the effects of different (re-)referencing schemes on SOs, spindles and ripples in the MTL. *Black*: linked mastoid reference, *blue*: white matter contact on the same electrode, *red*: bipolar reference of adjacent distal electrode contacts. Note that despite stepwise amplitude decreases, the morphology and number of detected events remains similar. **B**. For comparison, SO and spindle grand averages ± SEM are shown collapsed across scalp electrodes C3 and C4 (referenced to linked mastoids only).

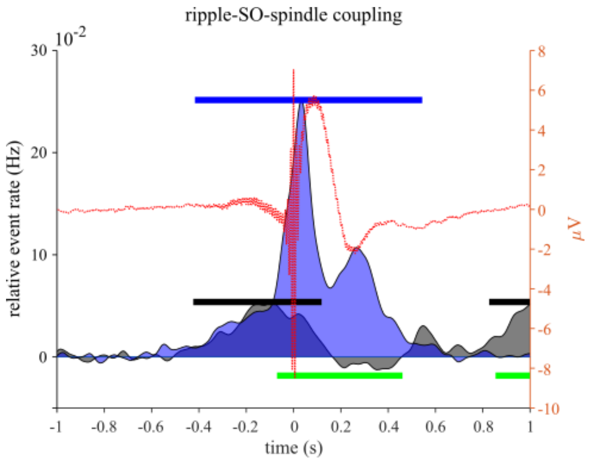

Figure S3. Spindle (blue) and SO (black) rates during ripples (dotted red line), relative to a pre-ripple baseline period. Note that unlike main Figure 2, event maximum times are plotted to better visualise SO up-states. Horizontal lines: blue … spindle occurrences vs. 0; black … SO occurrences vs. 0; green … spindle occurrences vs. SO occurrences, all P < .05 (corrected via cluster-based permutation test).

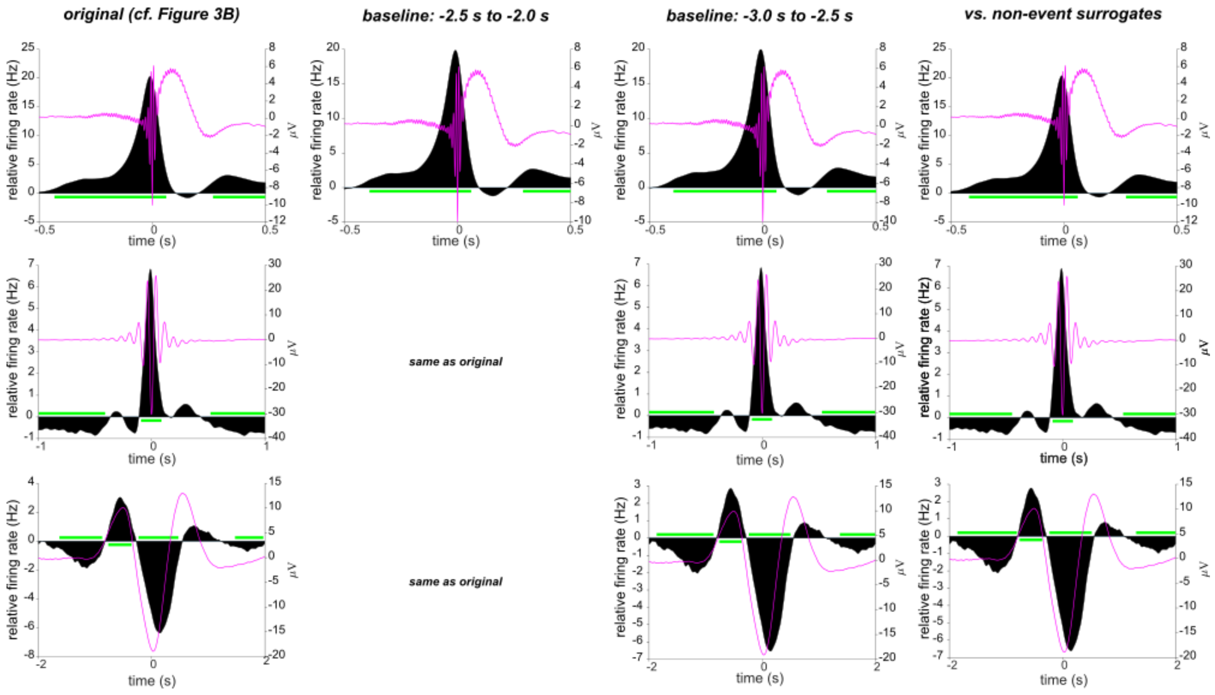

Figure S4.: Event-locked firing rates (FRs) after different baseline corrections (across columns). Top row: ripples, middle row: spindles, bottom row: SOs. Same plotting conventions as in main Figure 3B (horizontal green lines: $P < .05$, corrected via cluster-based permutation test).

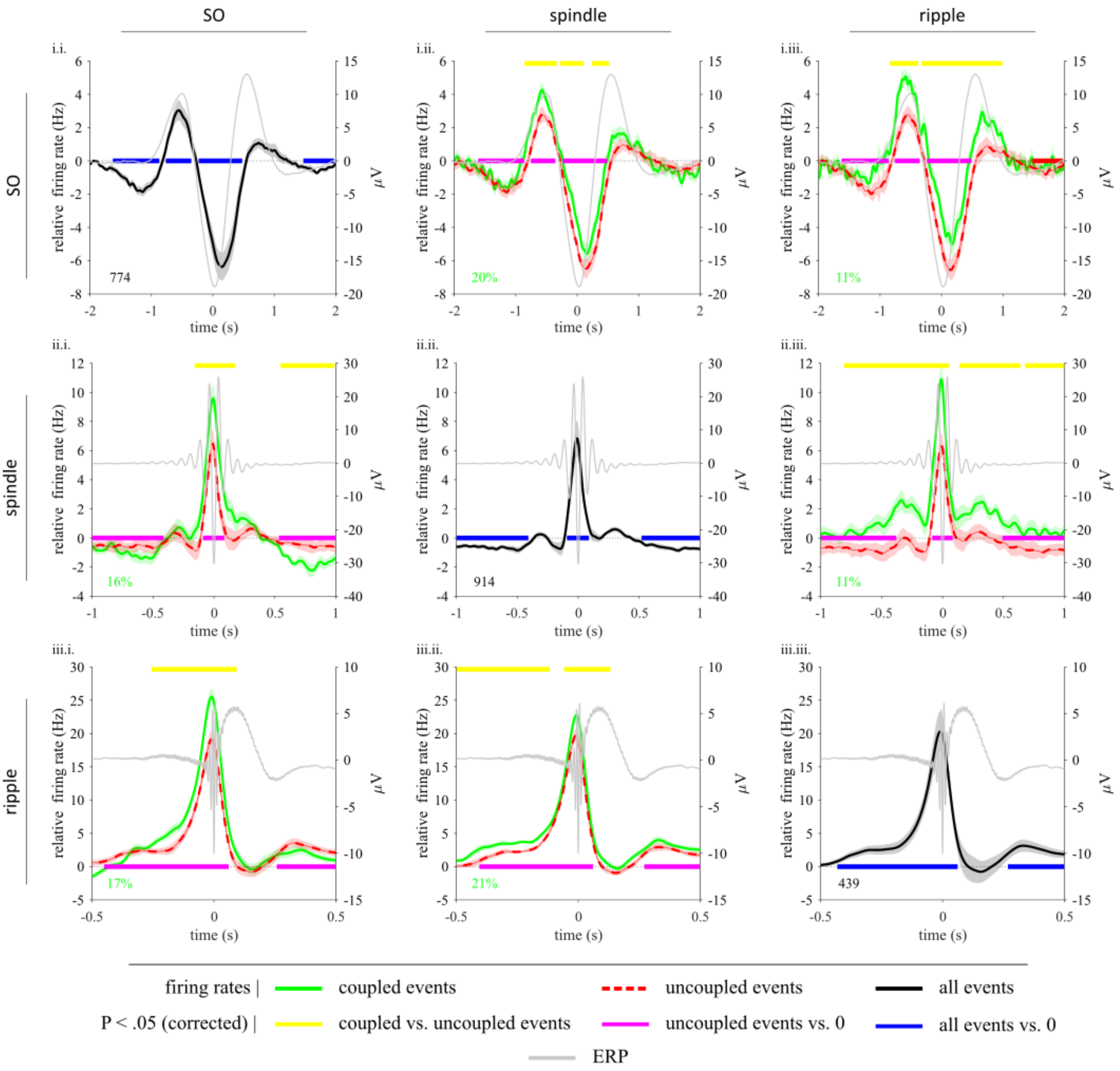

Figure S5. Event-locked neuronal firing rates (FRs) as a function of event contingencies (target event centre within ±1 s of seed event centre). *Rows*: seed events, *columns*: target events. *Diagonal*: all seed events (cf. main Figure 3A). Black lines represent mean baseline-corrected FR ± SEM of condition difference (coupled vs. uncoupled events). Grey lines represent grand average seed events (irrespective of target). Black numbers represent the number of events, averaged across contacts and sessions. Green numbers reflect percentage of coupled events (out of all seed events), averaged across contacts and sessions. Horizontal yellow, magenta and blue lines … P < .05 (corrected via cluster-based permutation test)

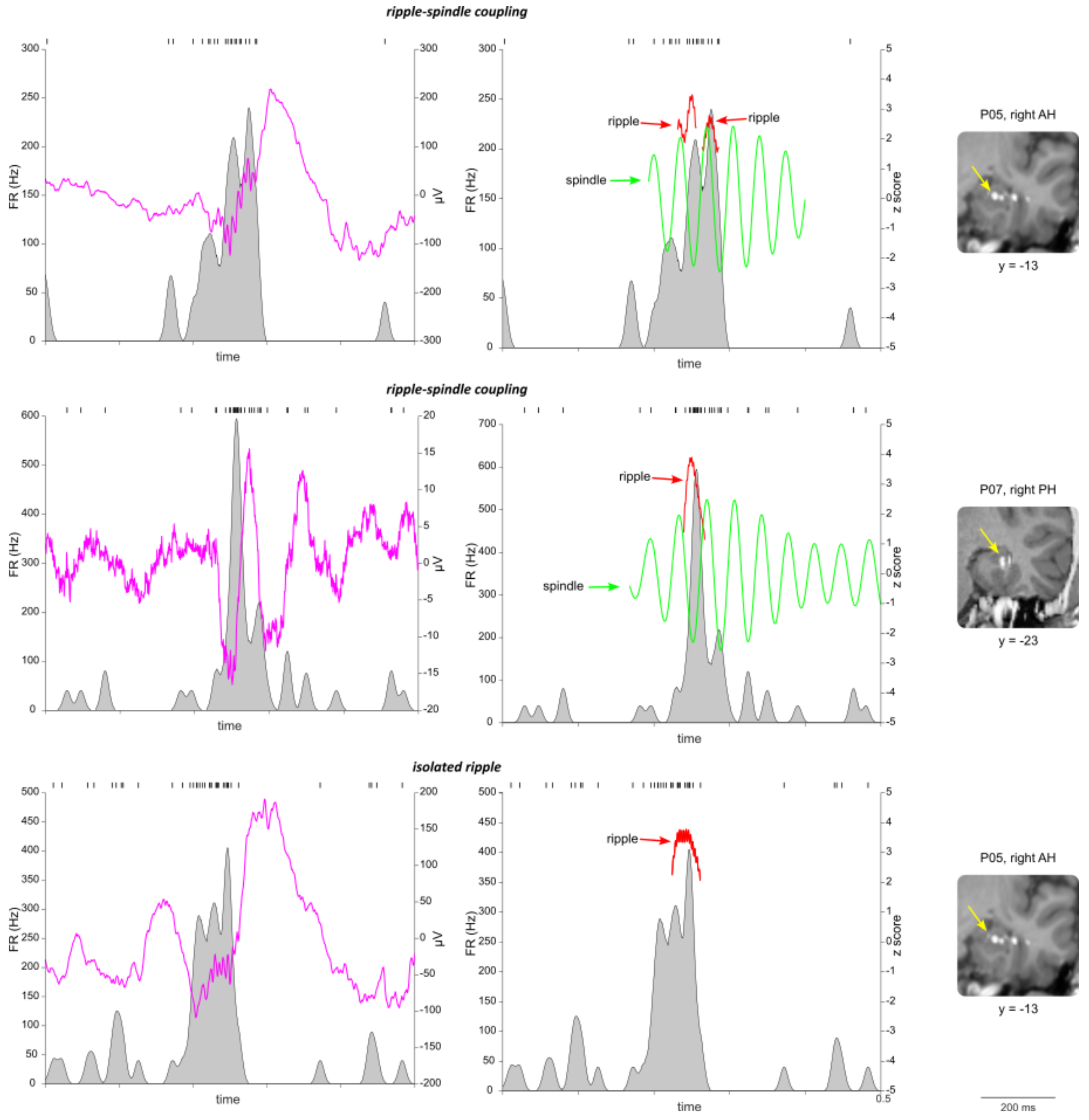

Figure S6A. Example raw (*left*) and bandpass filtered (*right*) EEG traces (right y-axis) and MUA firing rates (left y-axis). Pink: Raw EEG trace, green: z-scored, bandpass filtered spindle event (12-16 Hz), red: z-scored 20 ms RMS envelope of the bandpass filtered ripple event (80-120 Hz). Tick marks on top represent MUA firing rasters. Shaded grey areas are the corresponding instantaneous firing rate, obtained by applying a 100 ms Gaussian smoothing kernel. Right insets show anatomical locations in MNI space (AH = anterior hippocampus, PH = posterior hippocampus).

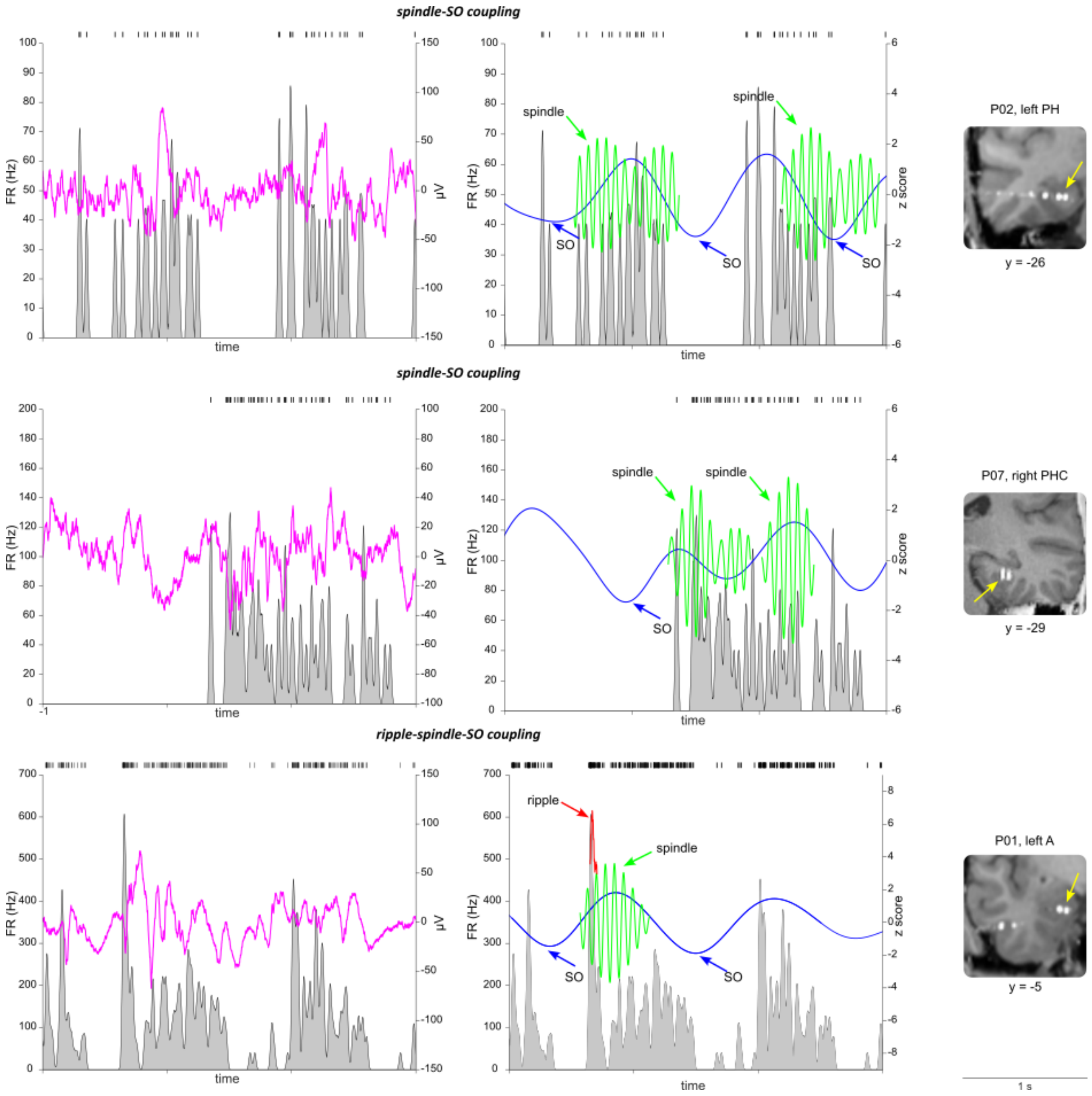

Figure S6B. Example raw (*left*) and bandpass-filtered (*right*) EEG traces (right y-axis) and MUA firing rates (left y-axis). Pink: Raw EEG trace, blue: z-scored, bandpass-filtered SO signal (.3-1.25 Hz), green: z-scored, bandpass filtered spindle event (12-16 Hz), red: z-scored 20 ms RMS envelope of the bandpass filtered ripple event (80-120 Hz). Tick marks on top represent MUA firing rasters. Shaded grey areas are the corresponding instantaneous firing rates, obtained by applying a 100 ms Gaussian smoothing kernel. Right insets show anatomical locations in MNI space (A = amygdala, PHC = parahippocampal cortex, PH = posterior hippocampus).

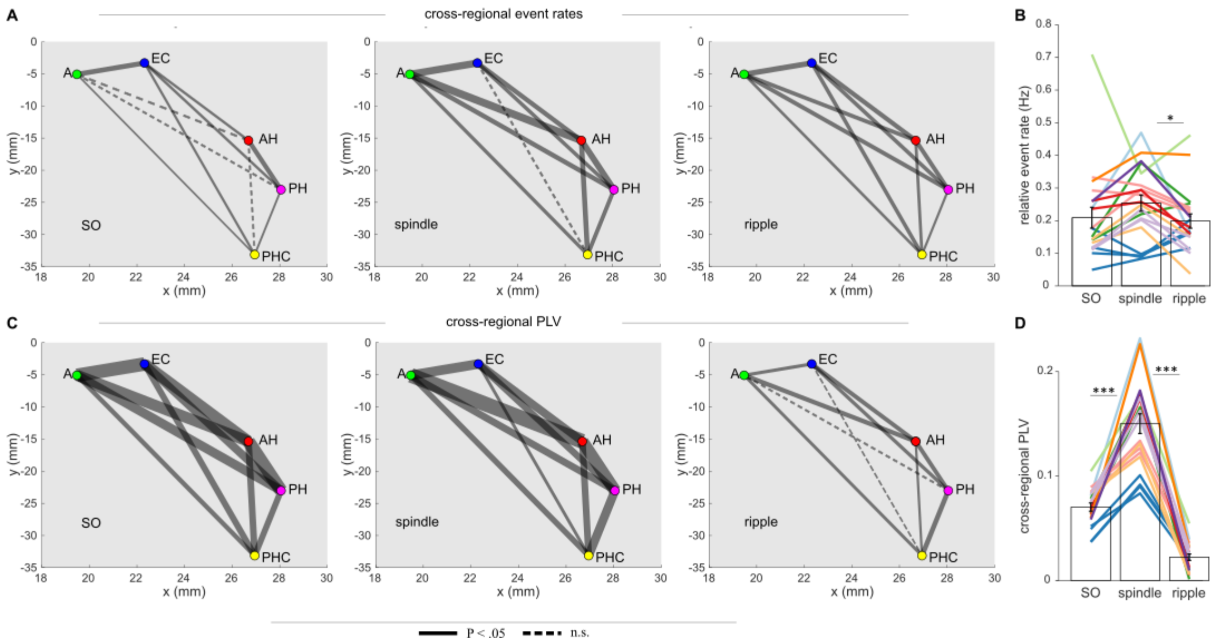

Figure S7. MTL network dynamics for all pairwise region combinations. **A.** Cross-regional event rates (co-occurrences, c.f., Figure 5B). **C.** Cross-regional phase locking values (PLVs, c.f., Figure 5C). Nodes reflect projections of MTL coordinates (MNI space) on the XY plane. Edge widths reflect effect sizes (t values of paired-samples t test) of bidirectional increases in event rates/PLV relative to a pre-event baseline. Solid line: significant bidirectional increase ($P < .05$, two-tailed, uncorrected). Dashed line: not significant **B** and **D**: All edges collapsed. Bars show mean ± SEM of conditions across sessions (N=20, recorded from 10 individuals). Individual lines represent individual sessions, with sessions from same participants grouped by colour. **B**. SO vs. spindle: $t(19) = 2.10$, $P = .050$; spindle vs. ripple: $t(19) = 2.24$, $P = .037$ (both two-sided paired-samples t tests). **D**. SO vs. spindle: $t(19) = 9.09$, $P = 2.37 \times 10^{-08}$; spindle vs. ripple: $t(19) = 11.75$, $P = 3.69 \times 10^{-10}$ (both two-sided paired-samples t tests) * … $P < .05$, *** … $P < .001$ (two-sided paired-samples t test).

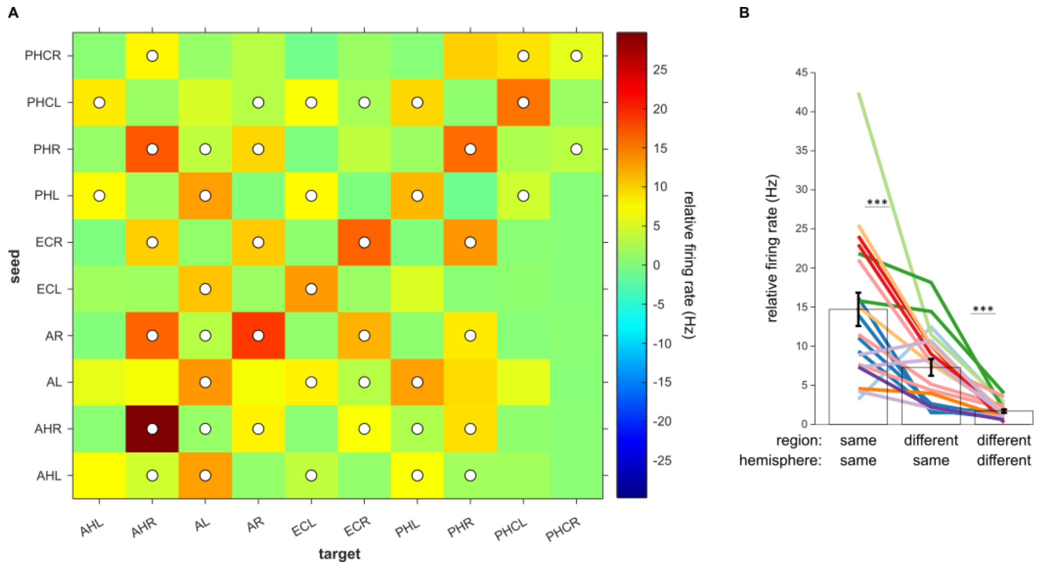

Figure S8. A. Ripple-locked cross-regional firing rate modulation relative to a pre-event baseline window (c.f. Figure 5D), separated by region and hemisphere. AH: anterior hippocampus, A: amygdala, EC: entorhinal cortex, PH: posterior hippocampus, PHC: parahippocampal cortex, L: left, R: right. White circles … $P < .05$ (two-sided, uncorrected). **B.** Comparison of (i) same region/same hemisphere combinations (c.f., Figure 3B *right*, but without weighing regional contributions to session means by the number of ripples), (ii) different region/same hemisphere combinations and (iii) different region/different hemisphere combinations. Individual lines

represent individual sessions, with sessions from same participants grouped by colour. Same region/same hemisphere vs. different region/same hemisphere: t(19) = 3.87, P = .001; different region/same hemisphere vs. different region/different hemisphere: t(19) = 5.65, P = 1.90 x 10^{-05} (both two-sided paired-samples t tests). *** … P ≤ .001 (two-sided paired-samples t test). For same region/same hemisphere combinations, a 2x5 repeated measures ANOVA with the factors Hemisphere (left, right) and Region (EC, A, AH, PH, PHC) yielded no main effects or interaction (all F < 2.83, P > .05).

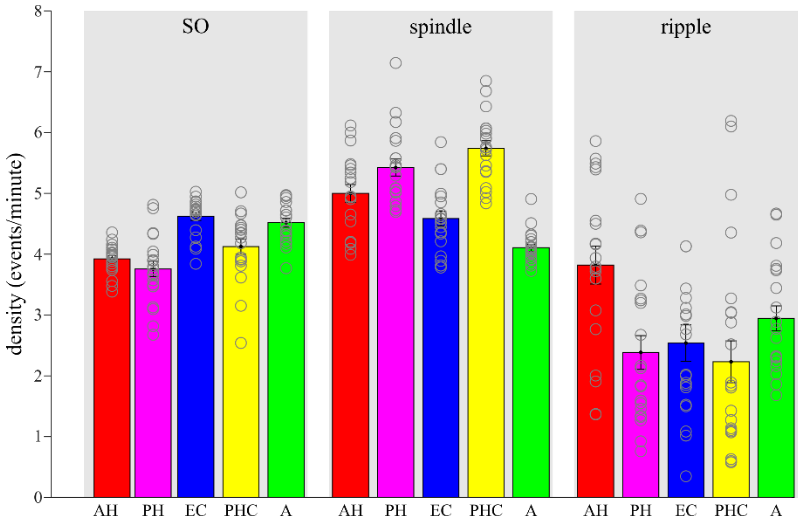

Figure S9. Event densities separated by MTL region. Bars represent mean ± SEM (N=20 sessions recorded from 10 individuals). Circles represent individual sessions. AH = anterior hippocampus, PH = posterior hippocampus, A = amygdala, EC = entorhinal cortex, PHC = parahippocampal cortex.

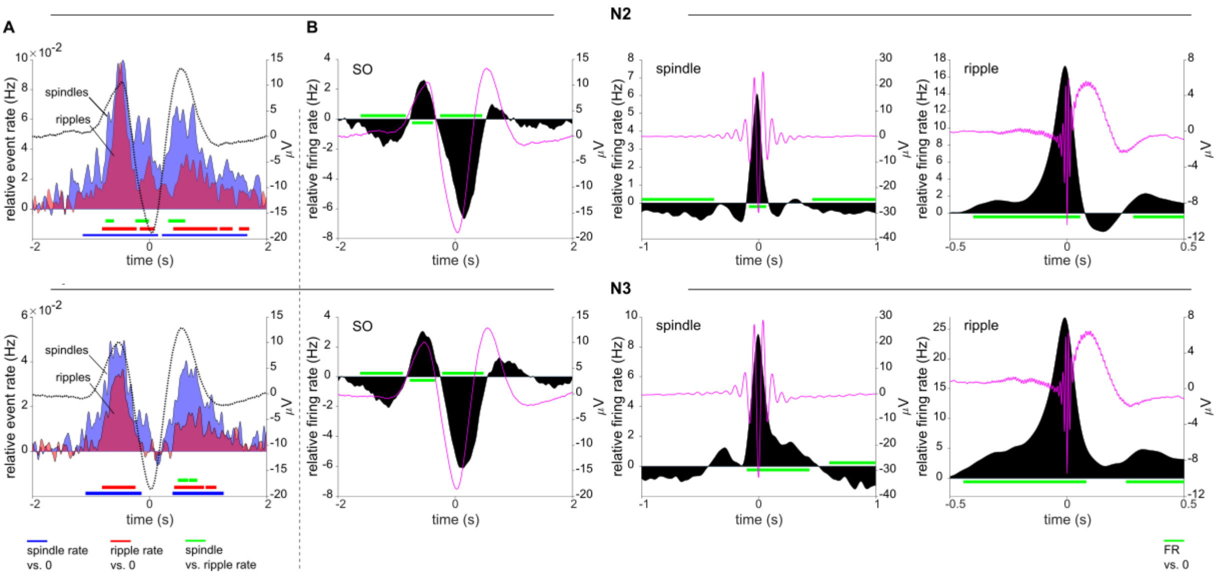

Figure S10. Main results shown separately for NREM stages N2 (*top*) and N3 (*bottom*). **A.** Coupling of spindles (blue) and ripples (red) to SO upstates. **B**. Modulation of MUA firing rates by SOs (*left*), spindles (*middle*) and ripples (*right*). All P < .05, corrected. Same plotting conventions as in Figures 2A and 3B.

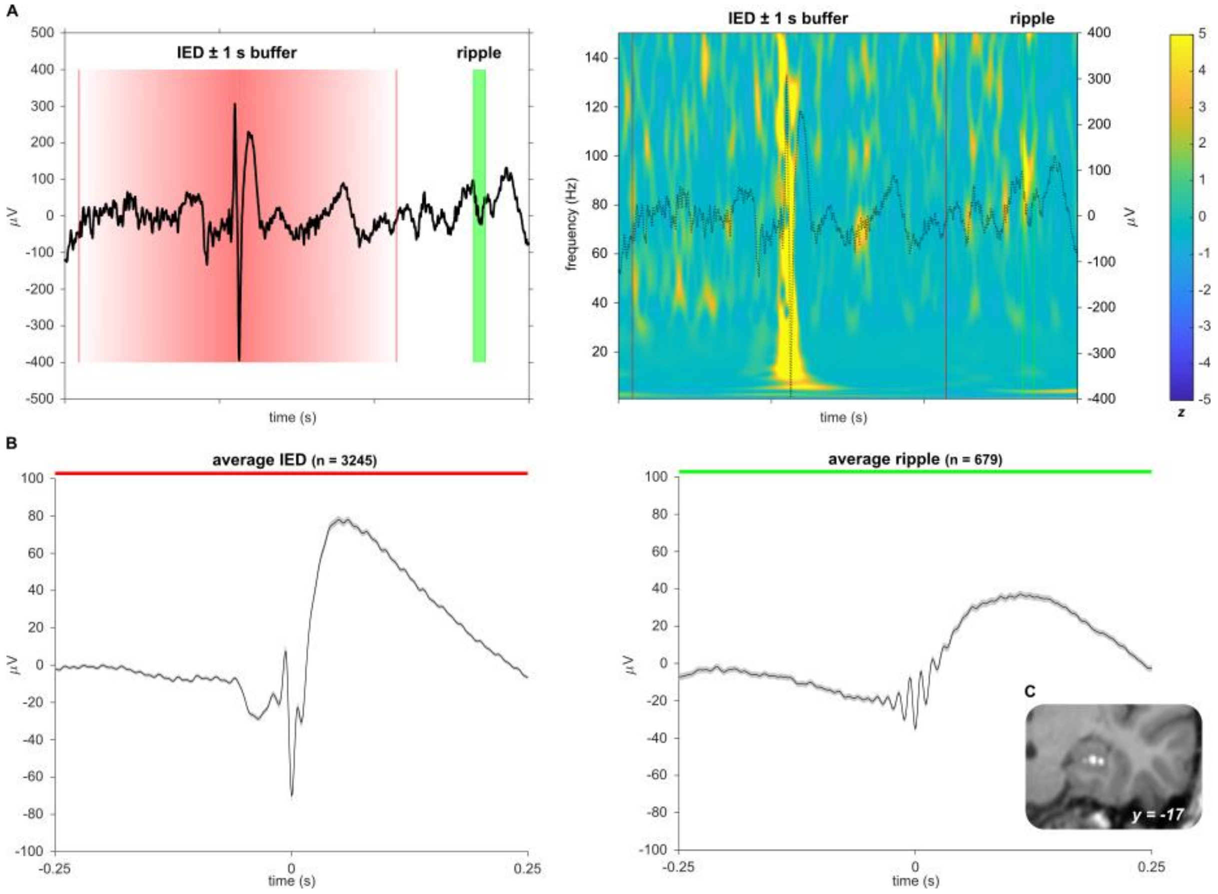

Figure S11. Pathological interictal epileptiform discharges (IED) and putatively physiological ripples detected by our pre-processing. Data from a right hippocampal contact shown in **C**. **A**. *left*: 3 s raw data sweep, showcasing a detected IED along with the +/- 1 s buffer period (*red*) and a detected ripple (*green*). *right*: time-frequency decomposition of the same data, z-scored across time (raw EEG trace superimposed, right y-axis). Note the circumscribed 80-100 Hz burst for the ripple as opposed to the broadband power increase during the IED. **B**. *left*: Average (+/- SEM) of all detected IEDs in this contact (n = 3245) after excluding physiological ripples. *right*: Average (+/- SEM) of all detected ripples in this contact (n = 679) after excluding IEDs/artefacts (as per our standard procedure).

	minutes	%
**N1**	95 (8)	18 (1)
**N2**	174 (13)	32 (2)
**N3**	76 (5)	15 (1)
**REM**	75 (8)	14 (1)
**WASO**	114 (15)	21 (3)

Table S1. Sleep architecture in minutes and percentages (mean and SEM across sessions, N=20 recorded from 10 individuals). WASO … wake after sleep onset.

	**NREM (minutes)**	**#ripples**	**#spindles**	**#SOs**	**epileptogenic zone**
P01S01	354	5657	13284	9080	unclear
P02S01	264	4997	10613	10227	right MTL
P02S02	199	3913	7510	6365	---
P02S03	250	4630	9895	8642	---
P02S04	291	5484	11631	10252	---
P03S01	269	8352	8386	7293	bilateral
P04S01	196	2273	3645	3149	bilateral
P04S02	152	2852	3719	2699	---
P05S01	275	6523	9697	8749	right MTL
P05S02	270	6170	9754	9281	---
P05S03	280	5297	10436	8976	---
P06S01	343	3761	13766	9898	left MTL
P06S02	326	3546	13837	9582	---
P07S01	265	2822	10770	9929	right MTL
P07S02	273	3049	12233	10245	---
P08S01	90	2168	4177	3823	left MTL
P09S01	249	4514	7737	7299	left MTL
P09S02	207	3543	6548	6383	---
P09S03	284	4203	8282	7906	---
P10S01	170	4061	6839	4984	bilateral
	**NREM (minutes)**	**#ripples**	**#spindles**	**#SOs**	
avg	250	4391	9138	7738	

Table S2. Number of minutes spent in NREM sleep separated by session; absolute numbers of detected ripples, spindles and slow oscillations separated by session; epileptogenic zone (if available) separated by patient.
