# Supplementary material for: How coupled slow oscillations, spindles and ripples coordinate neuronal processing and communication during human sleep
Source: Nat Neurosci. 2023 Jul 10;26(8):1429–37. doi: 10.1038/s41593-023-01381-w (PMC10400429; doi:10.1038/s41593-023-01381-w)
Supplement: Supplementary file 2 — Reporting Summary [file 41593_2023_1381_MOESM2_ESM.pdf]

nature portfolio

Corresponding author(s): Bernhard Staresina

Last updated by author(s): Jun 12, 2023

# Reporting Summary

Nature Portfolio wishes to improve the reproducibility of the work that we publish. This form provides structure for consistency and transparency in reporting. For further information on Nature Portfolio policies, see our Editorial Policies and the Editorial Policy Checklist.

## Statistics

For all statistical analyses, confirm that the following items are present in the figure legend, table legend, main text, or Methods section.

| n/a | Confirmed | |
|---|---|---|
| ☐ | ☒ | The exact sample size (*n*) for each experimental group/condition, given as a discrete number and unit of measurement |
| ☐ | ☒ | A statement on whether measurements were taken from distinct samples or whether the same sample was measured repeatedly |
| ☐ | ☒ | The statistical test(s) used AND whether they are one- or two-sided<br>*Only common tests should be described solely by name; describe more complex techniques in the Methods section.* |
| ☒ | ☐ | A description of all covariates tested |
| ☐ | ☒ | A description of any assumptions or corrections, such as tests of normality and adjustment for multiple comparisons |
| ☐ | ☒ | A full description of the statistical parameters including central tendency (e.g. means) or other basic estimates (e.g. regression coefficient) AND variation (e.g. standard deviation) or associated estimates of uncertainty (e.g. confidence intervals) |
| ☐ | ☒ | For null hypothesis testing, the test statistic (e.g. *F*, *t*, *r*) with confidence intervals, effect sizes, degrees of freedom and *P* value noted<br>*Give P values as exact values whenever suitable.* |
| ☒ | ☐ | For Bayesian analysis, information on the choice of priors and Markov chain Monte Carlo settings |
| ☒ | ☐ | For hierarchical and complex designs, identification of the appropriate level for tests and full reporting of outcomes |
| ☐ | ☒ | Estimates of effect sizes (e.g. Cohen's *d*, Pearson's *r*), indicating how they were calculated |

*Our web collection on statistics for biologists contains articles on many of the points above.*

## Software and code

Policy information about availability of computer code

| | |
|---|---|
| Data collection | Depth electrodes were furnished with bundles of nine microwires each (eight high-impedance recording electrodes, one low-impedance reference, AdTech, Racine, WI) protruding ~4 mm from the electrode tips. The differential signal from the microwires was amplified using a Neuralynx ATLAS system (Bozeman, MT). |
| Data analysis | Data were analysed in MATLAB (version R2022a) using FieldTrip functions (version 20201229) and SPM12. |

For manuscripts utilizing custom algorithms or software that are central to the research but not yet described in published literature, software must be made available to editors and reviewers. We strongly encourage code deposition in a community repository (e.g. GitHub). See the Nature Portfolio guidelines for submitting code & software for further information.

## Data

Policy information about availability of data

All manuscripts must include a data availability statement. This statement should provide the following information, where applicable:

- Accession codes, unique identifiers, or web links for publicly available datasets
- A description of any restrictions on data availability
- For clinical datasets or third party data, please ensure that the statement adheres to our policy

Data and analysis scripts to reproduce the main results are shared on the Open Science Framework (OSF, https://osf.io/8kevm/).

## Human research participants

Policy information about studies involving human research participants and Sex and Gender in Research.

| | |
|---|---|
| Reporting on sex and gender | Of the 10 participants included, 5 were female and 5 male as determined in the clinical workup. No sex- or gender-specific analyses were performed in this report. |
| Population characteristics | All participants were right-handed, with a mean age of 39.9 years and an age range of 20-62 years. No population characteristics were included as covariates in the current study. |
| Recruitment | The current sample is an opportunity sample, based on patient influx for epilepsy treatment. Patients were included in the study if they consented to microwire implantation and had concurrent macro- and scalp EEG recordings. |
| Ethics oversight | Medical Institutional Review Board at the University of Bonn. |

Note that full information on the approval of the study protocol must also be provided in the manuscript.

# Field-specific reporting

Please select the one below that is the best fit for your research. If you are not sure, read the appropriate sections before making your selection.

☒ Life sciences ☐ Behavioural & social sciences ☐ Ecological, evolutionary & environmental sciences

For a reference copy of the document with all sections, see nature.com/documents/nr-reporting-summary-flat.pdf

# Life sciences study design

All studies must disclose on these points even when the disclosure is negative.

| | |
|---|---|
| Sample size | Macro- and simultaneous microwire recordings were performed in 20 sessions from 10 participants, admitted to the Department of Epilepsy for pre-surgical monitoring in a 2.5 years time period. No a-priori sample-size calculation was performed. The number of recordings sessions comfortably complies with or exceeds current standards in human microwire recordings.<br><br>References:<br>Clemens, Z., et al. Temporal coupling of parahippocampal ripples, sleep spindles and slow oscillations in humans. Brain 130, 2868-2878 (2007).<br>Staresina, B.P., et al. Hierarchical nesting of slow oscillations, spindles and ripples in the human hippocampus during sleep. Nature neuroscience 18, 1679-1686 (2015).<br>Skelin, I., et al. Coupling between slow waves and sharp-wave ripples engages distributed neural activity during sleep in humans. Proceedings of the National Academy of Sciences 118, e2012075118 (2021).<br>Dickey, C.W., et al. Travelling spindles create necessary conditions for spike-timing-dependent plasticity in humans. Nature communications 12, 1-15 (2021). |
| Data exclusions | No data were excluded from the sample. |
| Replication | Findings are based on stringent statistical assessments, applying corrections for multiple comparisons where appropriate. No explicit attempts at replication were taken. |
| Randomization | The study contains no experimental groups. |
| Blinding | The analysis contained no experimental conditions, so blinding did not apply. |

# Reporting for specific materials, systems and methods

We require information from authors about some types of materials, experimental systems and methods used in many studies. Here, indicate whether each material, system or method listed is relevant to your study. If you are not sure if a list item applies to your research, read the appropriate section before selecting a response.

## Materials & experimental systems

| n/a | Involved in the study |
|---|---|
| ☒ | ☐ Antibodies |
| ☒ | ☐ Eukaryotic cell lines |
| ☒ | ☐ Palaeontology and archaeology |
| ☒ | ☐ Animals and other organisms |
| ☒ | ☐ Clinical data |
| ☒ | ☐ Dual use research of concern |

## Methods

| n/a | Involved in the study |
|---|---|
| ☒ | ☐ ChIP-seq |
| ☒ | ☐ Flow cytometry |
| ☒ | ☐ MRI-based neuroimaging |
